# Implementation of a Blind Quality Control Program in a Forensic Laboratory

**DOI:** 10.1111/1556-4029.14259

**Published:** 2019-12-24

**Authors:** Callan Hundl, Maddisen Neuman, Alicia Rairden, Preshious Rearden, Peter Stout

**Affiliations:** ^1^ Houston Forensic Science Center 500 Jefferson Street, 13^th^ Floor Houston TX 77002; ^2^ Center for Statistics and Applications in Forensic Evidence (CSAFE) 613 Morrill Road Ames IA 50011

**Keywords:** forensic science, blind quality control, blind testing, blind verification, intralaboratory comparison, proficiency testing, intralaboratory testing, intralaboratory proficiency

## Abstract

A blind quality control (QC) program was successfully developed and implemented in the Toxicology, Seized Drugs, Firearms, Latent Prints (Processing and Comparison), Forensic Biology, and Multimedia (Digital and Audio/Video) sections at the Houston Forensic Science Center (HFSC). The program was put into practice based on recommendations set forth in the 2009 National Academy of Sciences report and is conducted in addition to accreditation required annual proficiency tests. The blind QC program allows HFSC to test its entire quality management system and provides a real‐time assessment of the laboratory’s proficiency. To ensure the blind QC cases mimicked real casework, the workflow for each forensic discipline and their evidence submission processes were assessed prior to implementation. Samples are created and submitted by the HFSC Quality Division to whom the expected answer is known. Results from 2015 to 2018 show that of the 973 blind samples submitted, 901 were completed, and only 51 were discovered by analysts as being blind QC cases. Implementation data suggests that this type of program can be employed at other forensic laboratories.

Proficiency testing is a requirement of all accredited forensic science service providers. These tests provide insight into where errors occur and how they can be remediated, as well as serving as a means to assess training, methods, and interlaboratory differences 1. Open proficiency provides a tool to assess the performance of analytical steps but is limited for testing the entire process. Most proficiency tests are open, or declared, and as a result, forensic practitioners are aware they are being tested. This awareness often occurs because the test items do not resemble evidence and submissions received in the normal workflow of a forensic laboratory. Differences in packaging, paperwork, and other details like materials and case numbering clue analysts into the test environment. Also, proficiency tests do not account for differences in how casework is administered in the laboratory, such as whether analysts work individually or follow protocols that require consultation with another analyst, as with verification, or they are assisted by other analysts or supervisors. Despite these differences, analysts are asked to work test items as they would their routine casework and since proficiency tests are not blinded to analysts, accuracy rates may be inflated 2, 3.

One of the few analyses on proficiency testing, a national proficiency study conducted in 1977, indicated shortcomings to open proficiency testing 2. In the 1977 study, a sample set of nonblind proficiency tests were disseminated to laboratories along with a set of blind samples that resembled routine submissions. The findings suggested both false‐negative and false‐positive errors were more frequent with the blind samples.

Blind proficiency testing was recommended by the American Society of Crime Laboratory Directors/ Laboratory Accreditation Board (ASCLD/LAB) in the 2009 National Academy of Sciences (NAS) report 4. This recommendation states that forensic laboratories should conduct blind proficiency tests as a more precise test of an individual’s accuracy. In support of this recommendation, recent analysis of proficiency testing suggests that blind testing reduces error rates by as much as 46%, depending on the level of bias and potential for penalties received by the test taker 5. For penalties received by the test taker, the rate is reduced as much as 46% 5. Blind testing also capitalizes on the Hawthorne effect 6, the tendency for people to alter their behaviors when they know they are being monitored, by providing a scenario in which potential bias associated with proficiency tests is controlled and reduced.

Even though technology and instrumentation have improved since the 1977 study 2 was conducted, contemporary research continues to express a need for blind proficiency testing as a way to compliment open proficiency testing 2, 7, 8. In fact, blind proficiency tests have been suggested for forensic services since DNA was conceived for use in police work in the 1990s 8. While the value of open interlaboratory proficiency testing is not negated, laboratories that desire a more constant and unbiased way to continually monitor and improve the performance of their processes are encouraged to implement an intralaboratory blind proficiency testing program, in addition to proficiency tests 2, 7, 8.

In 2015, the Houston Forensic Science Center (HFSC) adopted the recommendations for blind proficiency testing by implementing a blind quality control (QC) program. HFSC is a local government corporation, overseen by a board of directors, that provides forensic services to the City of Houston and surrounding areas, primarily serving the Houston Police Department (HPD). Services are provided in seven technical disciplines: Forensic biology, toxicology, seized drugs, firearms, latent prints (processing and comparison), multimedia (digital forensics and audio/video), and a Crime Scene Unit, all of which are accredited to the International Organization for Standardization/International Electrotechnical Commission (ISO/IEC) 17025 standard by the American National Standards Institute (ANSI) National Accreditation Board (ANAB). The objective of HFSC’s blind QC program is to receive a real‐time assessment of analysis procedures, determine areas for improvement, and ensure that stakeholders are consistently receiving accurate and reliable results.

## Materials and Methods

The blind QC program is facilitated by HFSC’s Quality Division, which is organizationally separate from the laboratory sections and reports directly to executive management; thus, quality controls are prepared and introduced into the system by personnel not connected with the actual testing. The Toxicology, Seized Drugs, Firearms, Latent Prints (Processing and Comparison), Forensic Biology, and Multimedia (Digital and Audio/Video) sections all participate in the blind QC program at HFSC. Table 1 highlights implementation dates for blind testing in each of the six disciplines.

**Table 1:** Implementation date of blind quality control (blind QC) submissions by section.

Discipline	Implementation Month
Toxicology	September 2015
Firearms	
Blind verification	November 2015
Blind QC	December 2015
Seized drugs	December 2015
Forensic biology	October 2016
Latent prints	
Processing	October 2016
Comparison	November 2017
Multimedia	
Digital forensics	November 2017
Audio/video	June 2018

### Preparation of Blind Quality Control Samples

The blind QC cases are created to mimic real casework. Since the blind QCs appear like routine casework, they are not expected to receive any special treatment by the analyst, therefore, reducing the risk of introducing any intentional or unintentional bias into the analytical process. Prior to implementation, casework and evidence submissions were assessed for commonalities in evidence item types, packaging, offense types, and request wording for each forensic discipline. The information gained from the analysis of each discipline was used to mimic routine casework. Most of the casework received by HFSC is submitted by HPD, so blind QC samples are created to mimic HPD submissions in packaging, submission process, and request wording, as closely as possible.

Before creating the blind QC samples, a worksheet is prepared containing the following case information: agency case number, subject name, date of birth, driver’s license number (if applicable), address, and offense date, type, and time. The agency case numbers, incident numbers assigned by HPD, are generated through the HPD’s records management system (RMS) near the time of the listed offense date. Subject name and date of birth are generated using a fake name generator website, such as www.fakenamegenerator.com. The offense date and time are created close to the case submission date, and the offense type is based on the evidence type submitted. Toxicology cases require a driver’s license number, which is fabricated to mimic a Texas driver’s license number. Online mapping websites, such as Google Maps, are utilized to choose street addresses within Houston city limits.

After each blind test case is analyzed, HFSC Quality Division personnel records and tracks the relevant case and evidence information including submission dates, expected results, reported results, analytical techniques and/or instruments, assigned analysts, and report date. Since each section at HFSC receives unique items and casework for analysis, the procedures for blind QC creation and submission for each section is outlined below with the goal of highlighting the feasibility of implementing this program in other laboratories.

### Toxicology

The Toxicology section receives and analyzes blood and urine samples for the presence of drugs and alcohol. Because most cases received by the Toxicology section are blood samples from driving while intoxicated (DWI) cases, the DWI offense type is used to submit toxicology blind QCs. The toxicology samples are prepared using toxicology collection kits that HFSC supplies to HPD. The kit contains blood tubes, evidence seals, and a specimen ID form. For blind samples, three blood vials of known alcohol concentrations, purchased from an outside vendor, are placed into the kit. The blood tubes come labeled, prealiquoted, and spiked with known alcohol concentrations along with a certificate of analysis listing the target and theoretical concentrations for each analyte. The vendor label is removed prior to placement in the kit and the concentration recorded by the Quality Division staff member preparing the blind case for submission. The specimen ID form is populated with subject and case information as well as officer and collector (phlebotomist) information. The kit is then sealed with evidence seals and initialed with the submitting officer’s initials. If the reported alcohol concentration, plus or minus the uncertainty of measurement, encompasses the theoretical target concentration provided by the manufacturer, then the toxicology blind QCs are determined to be completed satisfactorily.

### Firearms

The Firearms section at HFSC conducts function testing of firearms, serial number restoration, and microscopic examination of bullets and cartridge casings to determine if the items were fired from the same firearm. Blind testing in the Firearms section is twofold: blind verifications and blind QCs. In a typical firearms examination, a primary examiner performs analysis on the evidence and documents the conclusions. Next, a verifier conducts a secondary examination during which the primary examiner’s case notes and conclusions are viewable. In a blind verification, the primary examiner’s case notes and conclusions are masked from the secondary examiner, consequently requiring an independent and unbiased examination of the same evidence. Blind verification cases are selected and assigned by the section management and conducted on real or blind QC cases. The Firearms section manager reviews and documents the initial conclusions after both examiners complete their analysis. After management review, the two examiners then discuss their individual results to determine if they reached the same conclusion or if further consultation is needed.

For firearms blind QCs, evidence such as fired or unfired bullets, casings, and/or bullet fragments are created by the Firearms section management. Fired evidence is created using firearms from the reference collection, which is a library of firearms used for parts and training, or firearms slated for destruction by HPD. The firearm(s) used to create the fired evidence may or may not be submitted as an item of evidence. Firearms management reviews the evidence prior to submission to determine the expected result as well as the results of the completed blind QCs in order to determine satisfactory completion.

### Seized Drugs

The Seized Drugs section at HFSC analyzes evidentiary substances for the presence of controlled substances. Blind QC drug evidence is created using either drug standards purchased from external vendors, substances that are not controlled, or drug evidence slated for destruction by HPD. The controlled substances submitted are drugs that are frequently encountered in the Houston area. To mimic normal drug evidence, the drug standards are mixed with diluents. For drug evidence slated for destruction, the samples are analyzed by the section supervisors prior to submission of the blind QCs. Samples are packaged within HPD‐issued narcotics envelopes that are filled out with fabricated case information. Seized drugs cases are determined to be completed satisfactorily if the expected substance is reported.

### Latent Prints

The Latent Prints section at HFSC performs two main tasks: latent print processing and latent print comparison. Latent print processing is the development of friction ridge detail on physical items of evidence. Latent print comparison is the observation and analysis of friction ridge detail to determine suitability for comparison as well as comparison of unknown prints to those from known sources taken under controlled conditions. Latent prints can also be searched through local, state, and federal databases against known record prints to determine potential sources of the fingerprint.

Houston Forensic Science Center was granted access by the local Automated Fingerprint Identification System (AFIS) to enter five sets of record prints (to include ten print exemplars) into the system under fictitious individual information, referred to as an alias. Because all HFSC staff is fingerprinted and registered into the state and federal databases upon hire, restricting searches to the local database allows HFSC staff to create evidence for cases with their fingerprints. The offense type used for latent prints blind QC cases is limited to property and nonviolent crimes, such as burglaries or auto thefts. This is due to the section’s standard procedure which calls for the HFSC latent print examiners to search or register latent prints from property or nonviolent crimes in the local database only. Limiting the offense type to property and nonviolent crimes prevents the prints submitted or developed from being searched and registered in the state or federal AFIS databases as required by the section standard operating procedure.

The blind QCs for latent print processing are created by first cleaning a nonporous item with an alcohol wipe to remove any pre‐existing latent prints. Items include anything that could have been handled by a subject at the scene of a crime, such as aluminum cans, tools, food containers, or cell phones. To deposit a known subject’s prints, the donor rubs their fingers over sebaceous residue and then handles items as one would normally handle such an item (i.e., hand placement as with routine use of a crowbar), submitted for latent processing. The identity of the person handling the item is recorded.

Due to the tenuous nature of friction ridge deposition, latent print processing cases cannot be guaranteed to produce prints. The latent prints processing blind QC cases are deemed completed satisfactorily if prints are developed. If latent prints are not developed, the case is deemed satisfactory if the correct sequential processing technique is used to process the sample.

For latent prints comparison blind QCs, latent fingerprint cards are created under fictitious names using fingerprints from staff members who volunteered to participate and be registered in the local AFIS. Since most of the evidence received for latent print comparison is latent lift cards, blind QC latent lift cards are created by depositing fingerprints onto a clean, smooth surface with varying degrees of deposition pressure and distortion like that resultant from twisting or movement of the fingers. Subsequently, the impressions are processed with black fingerprint powder, lifted with tape, and pasted onto the back of a blank HPD‐issued latent lift card. Blind QC latent lift cards are prepared in bulk in advance of submission. The agency case number, date lifted, offense type, address of collection and/or vehicle description, location of latent print(s) lifted, and name of submitter are recorded on the card. A sketch of the area where the latent was said to have been lifted is also drawn on the card.

The evaluation result is dependent on multiple factors, which includes the limitations of the AFIS systems, as well as the many possible outcomes of the latent print quality. If the latent print was determined by the latent print examiner to be of AFIS quality, it is searched in the local database. If the search results in a hit, referred to by HFSC as a preliminary AFIS association (PAA), the analysis is deemed satisfactory if the association is reported to the correct alias name of the person who created the prints. Cases that result in a PAA can be requested for full confirmation examination. Full confirmation cases are deemed satisfactory if an identification is reported to the correct alias. If a latent print is determined by the examiner to be of AFIS quality but searches in AFIS do not result in a hit, then the candidate list generated by that search is later reviewed by the Latent Prints section manager or technical leader to determine whether the donor’s alias record print was on the candidate list or not. Blind cases are determined to be completed satisfactorily if the donor candidate alias is not contained within the candidate list. Low quality prints, or prints with no ridge detail, are expected to be reported as “no latent of value,” “no ridge detail,” or “not AFIS quality.”

### Forensic Biology

The Forensic Biology section at HFSC collects and preserves trace evidence and potential contact DNA evidence, as well as identifies bodily fluids to analyze for DNA. Forensic biology blind QC evidence is created using known samples of saliva from HFSC staff volunteers or bodily fluid samples purchased from an external vendor. The Forensic Biology section is required to cross reference any unknown DNA profiles generated in a case against an internal database which includes HFSC staff and externally purchased DNA profiles. The internal database prevents blind QC profiles from being entered into the Combined DNA Index System (CODIS), but it also limits the scope of the Blind QC Program in the Forensic Biology section to analysis and interpretation.

Known, single source specimens of DNA are used to create contact, blood, and buccal swabs or the samples are placed directly onto items that might have been used in the commission of a crime (e.g., tools or knives). Preventive measures such as wearing appropriate personal protective equipment (PPE), decontaminating the workspaces before and after preparation, and preparing samples under a fume hood are taken when preparing the DNA cases. Forensic biology evidence items are prepared in advance and stored in a manner to prevent contamination. Forensic biology blind QC cases with sufficient DNA available on the evidence items are deemed satisfactory if the DNA profile generated is consistent with the single source DNA profile of the known contributor. In some instances, forensic biology cases did not generate sufficient DNA to conduct further analysis. Typically, this occurs with cases created with contact DNA samples. Like latent prints, forensic biology cases cannot be guaranteed to generate expected results, such as a DNA profile, since the amount of DNA deposited onto an item is variable.

### Multimedia

The Multimedia section at HFSC performs two main tasks: digital forensics and audio/video analysis. Digital forensics analysis includes examination of media, such as cell phones and computers, in order to perform data extraction. Audio/video analysis may include audio or video/image enhancement, format conversion, producing video segments, and rendering still images from a video.

Blind QC items for digital analysis are obtained by purchasing new or used mobile devices (e.g., cell phones, tablets, and media storage). If purchased new, the devices are used as normal to generate data usage, such as text messages, internet searches, phone calls, and emails. If the contents of the devices are unknown, the items are analyzed by section management, prior to submission, to determine the expected results from the data extraction. Digital forensics blind QCs are determined to be completed satisfactorily if the extracted data provided by the analyst is consistent with the request for analysis, as well as the information available on the device.

Blind QC items for audio/video analysis are created by obtaining audio or video footage from a security camera, cell phone, or other media device. The footage is viewed prior to submission to determine the appropriate request type based on the contents on the device. The audio or video is downloaded onto a CD or flash drive which is submitted for analysis. Audio/video blind QCs are determined to be completed satisfactorily if the data provided by the analyst fulfills the request.

### Blind QC Submission Process

The goal of the blind submission process is to mimic the submission of an item by an officer to the HPD Property Room. On the day of submission, case information is entered into HPD’s Evidence Management System (EMS). The blind QCs are taken to the HPD Property Room, by HFSC’s evidence technicians or Quality Division personnel, where an HPD barcode label for each item is printed and applied to the outer packaging. In accordance with HPD’s drug submission process, seized drugs blind QCs are delivered to centralized evidence receiving (CER), the narcotics intake portion of HPD’s Property Division.

Just like with real casework, a request for analysis is made on all blind QC items except for toxicology kits and latent print lift cards, which, through an established agreement with HPD, are automatically requested for analysis and transported back to HFSC the same day. Other evidence items are stored at the property room until requested for analysis, which is typical of real evidence. Since the requestor’s name is visible to the analyst in the laboratory information management system (LIMS), the request needs to be submitted by an HPD officer. At the start of the blind QC program, the Quality Division emailed an HPD officer to ask them to submit a request for analysis. In 2017, HFSC transitioned to a new LIMS that allows samples to be blinded to everyone but the Quality Division. A request portal that works in conjunction with LIMS was built with customizable features that benefit the blind program. One feature allows Quality Division personnel to submit a request on behalf of another person. To utilize this feature, several HPD officers granted HFSC permission to submit requests on their behalf. For analysts assigned a blind QC, the case appears to have been submitted by a real officer; however, the request was created by a member of the Quality Division on behalf of the officer. The officer is subsequently notified by the quality staff submitter that a test case was submitted with their officer information. This notification serves to prevent the officer from revealing the test scenario by way of nonrecognition of the request if contacted by HFSC analysts with follow‐up questions.

The assignment of blind cases to HFSC analysts is random except for the Latent Prints and Seized Drugs sections. In the Latent Prints section, management assigns most of the comparison cases to avoid the examiners receiving multiple cases with the same alias. However, to truly submit blind samples, there are instances where latent prints management is not notified, and cases are randomly assigned. In seized drugs, cases are assigned by supervisors to nonadjacent analysts. These nonadjacent assignments were initiated to reduce the chances of the analysts noticing the similarities in test scenarios, which happened frequently at the start of the blind program.

Once the blind program was established in each section, a submission rate goal was selected. By 2018, the targeted submission rate for each discipline was 5% of monthly completed casework. The target is calculated by taking the average number of cases completed per month for the previous year, multiplied by 0.05. The 5% target allows for a manageable submission rate that does not overwhelm the discipline or burden their caseload, while building a sample population that could be used to calculate error rates in the future. The 5% target submission goal for 2018, in each section, is outlined in Table 2.

**Table 2:** Number of blind quality control (blind QC) samples submitted per month in each forensic discipline in 2018.

Forensic Discipline	Blind QCs Submitted/Month*
Toxicology	14
Firearms	
Blind verification	1
Blind QC	1
Seized drugs	30
Forensic biology	10
Latent prints	
Processing	3
Comparison	10
Multimedia	
Digital forensics	1
Audio/video	1

* Approximately 5% of section casework completed per month during 2017.

## Results

A total of 973 blind QC cases were submitted into the workflow of the technical disciplines at HFSC from September 2015 to December 31, 2018. Analysis was completed on 901 of the submitted cases (the remaining 72 submitted cases were completed after December 31, 2018 and are not discussed within the scope of these results). Of the 901 completed cases, all were completed satisfactorily, and 51 were discovered by analysts as being blind QC cases. Refer to Table 3 for a section breakdown of blind QCs submitted, analyzed, and discovered during this time period.

**Table 3:** Number of blind quality control (blind QC) samples submitted, completed, and discovered from implementation date to December 2018.

Forensic Disciplines	Blind QCs Submitted	Blind QCs Completed	Blind QCs Discovered as Blinds
Toxicology	396	381	13
Firearms			
Blind verification	41	40	n/a*
Blind QC	26	25	3
Seized drugs	291	284	31
Forensic biology	36	16	0
Latent prints			
Processing	44	34	3
Comparison	120	107	1
Multimedia			
Digital forensics	12	10	0
Audio/video	7	4	0
Total	973	901	51

* Analysts were aware that they were participating in a blind verification.

The reasons the blinds were discovered varied across the forensic disciplines. Some toxicology blind QCs were discovered because the handwriting on the submission forms was too neat, which indicated to an analyst that the form was not filled out by a police officer. In another instance, toxicology blind QCs were discovered because several toxicology kits with consecutive agency case numbers (indicating that the cases were created around the same time) were submitted at the same time and the blood tubes from these kits were placed in the same batch for analysis.

Seized drugs blind QCs were also discovered due to minor inconsistencies in the submission process and through samples that did not mimic casework. In one instance, two analysts, who work in close proximity, concurrently received an aliquot of the same sample for analysis. Upon reviewing the Fourier‐transform infrared spectroscopy (FTIR) spectra, the analysts noticed that the spectra were almost identical, subsequently discovering the blind QCs.

Firearms blind QCs were also discovered for a variety of reasons. For example, an examiner recognized that the gun submitted was from their reference collection. In other cases, the packaging was not consistent with normal casework. Another examiner correctly identified a blind QC sample by stating that fired evidence looked “too clean.” Once, while test firing a submitted gun, an examiner believed the gun “smelled familiar” which led her to think it was a blind QC. Surprisingly, this examiner in fact had never analyzed this particular firearm but correctly identified that this case was a blind QC.

The three latent prints processing blind QC samples were discovered due to the prints being “too good” or that the item of evidence had not been handled in a natural manner. Specifically, an analyst was given a crowbar to process, and prints were found in areas where a crowbar handled normally would not have prints. The latent prints comparison blind QC was discovered because a request for confirmation was made on an item; however, the Quality Division had retrieved the item from the HPD Property Room and was present on the chain of custody. The assigned examiner realized the case involved a blind QC because the item’s chain of custody showed the evidence as being in possession of the Quality Division.

Any unanticipated blind QC result has been due to preparation issues, not analysis. For example, contamination during preparation of drug samples by the Quality Division led to an unexpected peak of methamphetamine in a cocaine sample. The analyst reported that the sample contained methamphetamine and cocaine, when the Quality Division was expecting the sample to yield a result containing only cocaine. In another blind sample, a cigarette dipped in phencyclidine (PCP) had been submitted for analysis for a second time; however, there was not enough PCP sample on the cigarette to detect during analysis. This was likely because the PCP sample was purchased as a reference sample and the concentration was very low. Both the original and second submission samples were reanalyzed by a supervisor to confirm the results.

Reviews of completed cases led to preventive actions in two instances. In one digital forensics blind QC, the analyst performed a manual extraction of text messages from a cellular phone; however, due to the format of the text messages, the messages were only partially captured. The digital forensics’ standard operating procedure (SOP) was revised to include a more descriptive procedure for manual extractions. In the other instance, a seized drugs case was submitted for analysis along with a request on the item’s original packaging for latent print processing. The narcotics submission form has a check box that the officer marks when a latent print processing request is needed. The Seized Drugs supervisors are responsible for looking for the check mark. If the form is checked, then the section supervisor creates a latent print processing request in LIMS. A secondary check is performed by the analysts, before analysis, to verify that the request was created. On the blind QC submitted to seized drugs, the supervisor and the assigned analyst missed the checked box and proceeded with analysis, voiding the request for latent print processing. A third level of verification was added for these request types, after this incident. Now, the evidence technicians who deliver the cases to the section also look for the checked boxes and make sure a request has been made for latent print processing.

Of the 40 completed firearms blind verifications, eight have led to consultations between the primary and the secondary examiners. A consultation occurs when the primary and secondary examiners reach different conclusions between items of evidence. In the consultation, the two examiners discuss the justifications utilized to reach their individual conclusions until a common conclusion is reached. If a common conclusion cannot be reached after a consultation, this scenario is then escalated to a conflict. A conflict requires a third examiner to conduct an independent analysis. To date, there have been no conflicts in a blind verification or a blind QC case. Furthermore, in the Latent Prints section, six of the blind QC cases that resulted in PAAs were requested for full confirmation. All six cases resulted in correct identifications to the alias donors.

## Discussion and Conclusions

### Cost

The cost of intralaboratory testing has been cited as a major impediment to adopting blind testing programs in forensic laboratories 7, 9, 10. While the blind QC program at HFSC did come with significant up‐front supply costs, not all supplies were costly. With the exception of toxicology blind QCs, the annual cost of sample preparation is minimal compared with the cost of traditional open proficiency tests. The estimated costs of supplies for blinds compared with the estimated costs for traditional open proficiency tests are listed in Table 4.

**Table 4:** Approximate costs of blind quality control (blind QC) samples and externally purchased proficiency tests (PTs) samples per year.

Forensic Disciplines	Cost of Supplies for Blind QC Samples				Cost of External PTs*			
	2015	2016	2017	2018	2015	2016	2017	2018
Toxicology	$16,000	$122	$16,716	$28,901	$1950	$2010	$1720	$1765
Firearms	$0	$0	$0	$0	$2790	$2580	$2300	$2245
Seized drugs	$0	$0	$5300	$165	$3240	$2960	$3230	$3060
Forensic biology	n/a	$0	$1840	$0	$7732	$8633	$8608	$8262
Latent prints	n/a	$0	$0	$20	$5340	$6520	$6130	$6060
Digital forensics	n/a	n/a	$0	$378	$1898	$2490	$2786	$2490
Audio/video	n/a	n/a	n/a	$221	$1750	$3150	$4550	$4125
Miscellaneous	n/a	n/a	$1210	$334	n/a	n/a	n/a	n/a

* An external PT was typically purchased for each analyst during these years.

Toxicology is the most expensive blind quality control program to facilitate, but also the most robust blind QC program at HFSC 11. Approximately $16,000 was spent on purchasing the initial lot of 400 blood tubes in 2015. This initial lot lasted through 2016, and additional samples were purchased in 2017 and 2018. The additional blood tubes were purchased to meet the 5% caseload benchmark, due to an increase in section casework. HFSC also started to supply stakeholders with toxicology kits containing three blood tubes, as opposed to previous kit versions containing two, which resulted in the purchase of additional tubes that subsequently contributed to the 2018 cost increase.

Compared with expenses incurred for the toxicology blind QCs, the supply costs for the other forensic disciplines are relatively low. DNA samples for forensic biology blind QCs, like the blood tubes, are purchased externally as needed, and buccal swabs or contact swabs from HFSC personnel are submitted at minimal cost. The cost of seized drugs blind QCs are minimal because they are created using HPD evidence that is slated for destruction. Prior to HFSC obtaining seized drugs evidence from HPD, purchasing drug standards (e.g., cocaine and methamphetamine) was a significant up‐front cost. Now, the only cost for seized drugs blind QCs involves the purchasing of paraphernalia found in typical drug cases submitted by HPD.

Similarly, costs in latent prints, digital forensics, and audio/video are relatively low, due to the types of evidence submitted (e.g., assorted tools, used tablets, and video recording equipment) and the frequency of case submission. All firearms evidence is created in‐house at little to no additional cost to HFSC. Other expenses include PPE, packaging supplies, and items that can be submitted as evidence in more than one discipline.

Time and labor are other resources that factor into the cost of a blind QC program. Quality Division personnel at HFSC spend several hours per week preparing and submitting blind QC samples and recording and tracking results, in addition to performing their normal quality duties. The blind program is facilitated by five Quality Division personnel, but most program duties are the primary responsibility of one person. This suggests that a minimum of two full time staff members, fully dedicated to blinds, is needed to maintain a blind QC program. Personnel cost associated with the blind testing is not reported since HFSC staff is required, per accreditation requirements, to demonstrate competency and proficiency in their field of employment. HFSC considers the blind QC program a means to evaluate staff performance in these areas.

### Implementation Challenges

The first blind QCs were introduced in toxicology in 2015. The lessons learned from implementing the blind QC program in toxicology helped to provide a road map for employing the program in other sections. As shown in Table 1, sections were integrated into the program over several years.

One of greatest challenges to implementing the blind QC program was creating evidence items that closely mimicked routine casework. Analysts are accustomed to the specifics of their casework and notice minor discrepancies. For example, at the start of the program, analysts were able to detect blind QCs due to the neat penmanship on the evidence submission forms and packaging. As a result, the Quality Division began to disguise their handwriting as much as possible by writing messier or using a nondominant hand. Packaging also posed a challenge because of the specific ways the HPD Property Room packages items; any packaging that was inconsistent with the HPD packaging process was easily detected by the analysts. This discrepancy required the Quality Division to conduct further research and observations into routine evidence packaging procedures to keep the cases blind.

Additionally, using evidence items that appear authentic to the analysts was essential to masking blinds. When the program was first implemented in seized drugs, the Quality Division was limited in the substances they could obtain for use as drug evidence. Drug standards purchased from a vendor were used, but these substances did not mimic street drugs commonly analyzed by the Seized Drugs section. The analyst familiarity with controlled substances common to the Houston area and the drug appearance made nonconforming blind samples easily detectable. Similarly, blind samples were discovered in firearms because the examiners were familiar with the guns in the reference collection used to create the blind QC cases. To overcome these challenges, HFSC leveraged its positive working relationship with HPD to obtain drug evidence, mobile devices, and firearms slated for destruction for use in the blind QC program. This partnership allowed the Quality Division to create more realistic blind samples and helped to guarantee sustainability of the blind QC program.

All forensic laboratories struggle with acceptable quality of evidence submitted. The blind program has made this apparent within the system at HFSC. Making sufficiently authentic materials that are representative of typical cases will always be a challenge for blind testing. But the extent to which devising a means of emulating common mistakes in evidence has been necessary suggests errors in packaging and sample identification happen far too often, and significant improvements in evidence management are needed.

Another factor to consider in the implementation of a blind QC program is analyst awareness and engagement. While many of HFSC’s initial cases were detected due to inconsistencies with normal casework, HFSC did not want the analysts to not report the discovered blinds, as this would defeat the purpose of the program. Consequently, an incentive was offered to anyone that detected a blind case. This incentive created a hyperawareness of the program among analysts. Analysts are awarded a Starbucks gift card for correctly discovering a blind case, while a much smaller monetary penalty ($1) is issued for incorrectly identifying a blind. When analysts believe they have found a blind QC, they must provide the reasons why they believe the case is a blind submission. Information gained from the correct identification of the blind test scenario is used to improve the quality of the blind samples and submission process.

Gaining buy‐in from the stakeholders (e.g., submitting agency, district attorney’s office, and staff) is one of the biggest challenges related to maintaining a blind QC program. The stakeholders’ participation and support of the program, as well as their understanding of how it benefits them as customers, is critical to its success. The partnership between HFSC and HPD, regarding obtaining evidence slated for destruction as well as using RMS and EMS and making analysis requests on behalf of officers, illustrates the importance of stakeholder buy‐in. The HFSC President and CEO works closely with HPD and the City of Houston to form relationships to facilitate collaboration and gain continual support for the program.

### Benefits

HFSC continuously benefits from the blind QC program. Blind testing at HFSC has shown to be a complementary and advantageous quality control tool that can be used to objectively evaluate the laboratory system and more closely mimic real casework. It has allowed HFSC to appropriately gauge the proficiency of its staff and the procedures used during analysis. The program tests the entire quality management system, reveals opportunities for improvement throughout the laboratory, and fills in the gaps in proficiency testing. For example, the blind evidence samples more closely mimic the types of items typically received by the laboratory in the way they are packaged, how they are handled through the submission process, and the types of analyses performed. Upon submission, the blind QC samples are transferred and stored in the same manner and under the same conditions as real evidence. If any unexpected changes in storage conditions were to happen, such as a refrigerator failure, then the results of the blind QC test cases affected by the temperature change can be evaluated to determine the impact on real evidence samples.

Blind QC samples submitted through the normal workflow can also provide a more appropriate gauge on the effectiveness of company policies and procedures. Blind samples allow for the simulation of real‐life case scenarios. For example, HFSC has a policy in place for processing multi‐disciplinary requests, which occurs when more than one test is requested on an evidence item, such as a request for latent prints processing and DNA analysis. Requesting more than one analysis on a single blind QC item enables HFSC to see if the requests are being processed in the appropriate order according to company policy (i.e., DNA will always be the priority analysis). Unlike proficiency tests, blind QCs can flow through the different forensic disciplines, allowing the Quality Division to observe how the items move through each section’s workflow; thus, determining if samples were handled and analyzed appropriately and in the correct order.

### Recommendations

The most significant factors that have contributed to the successful implementation of the HFSC blind QC program have been 1) the positive relationships and collaborations with stakeholders, 2) dynamic analyst engagement, 3) a gradual implementation rate, and 4) the ability of blind QC samples to effectively mimic casework. Addressing these issues is key to building a blind QC program, as discussed below.

#### Collaborations and Stakeholders

Forming partnerships with external collaborators and stakeholders is key to implementing and maintaining a blind QC program. Processes like evidence submission and entering analytical data into large algorithmic search databases, like AFIS, will require most laboratories to partner with law enforcement agencies. This type of partnership is exemplified in HFSC’s working relationship with HPD and the local AFIS administrator. Permission to utilize HPD’s records and evidence management software and the local AFIS database has greatly improved HFSC’s ability to mimic casework. Without access to these systems, HFSC would not be able to generate agency case numbers and submit cases in a realistic manner, which is an integral part of the blind QC program. Another benefit to having a partnership with law enforcement is the ability to use evidence slated for destruction, which can help lower the cost of blind supplies. Engaging stakeholders, both internally and externally, as early and as often as possible, can help drive the long‐term success of a blind QC program.

#### Analyst Engagement

Implementing blind testing requires both positive analyst engagement and active managerial support. Having management serve as champions for the blind program sets the tone for accepting blind QCs as a normal quality practice. If analysts buy into the program, they are more likely to be invested in the success of the program. For instance, when an analyst helps to identify blind samples, it provides insight into the handling of actual evidence items and discrepancies encountered during routine casework. When analysts openly communicate the discovery of blind samples, that information can be used to improve the blind QC process. Additionally, the blind QC program provides analysts with empirically validated data to support their court testimony.

#### Implementation Rate

Implementing a blind QC program can be logistically challenging and requires time, effort, and money. Many of these issues can be mitigated through the sharing of knowledge and resources. As more laboratories share their SOPs, challenges arising from sample creation, case submission, and lessons learned can be alleviated and/or overcome. Submission and implementation rates are other factors to consider when creating a blind QC program. The blind QC program at HFSC was gradually implemented, and the blind samples initially submitted in small batches. The disciplines added at later stages benefitted from the lessons learned from the early adopters of the blind program. This measured approach made it easier to identify and troubleshoot issues related to submissions and analytical procedures.

#### Blind QC Samples

The most fundamental goal of any blind QC program is to ensure that blind QC samples not only mimic routine evidence in appearance and submission, but also in the way they move through each section’s workflow. Prior to beginning a blind QC program, it is important to observe and research the forensic disciplines to learn the details of their case workflow. Details like evidence types, logistics, and procedures are vital when creating evidence samples and submitting requests that are believable as true casework. Discrepancies in chain of custody or submitting agency and requestor information can be used to identify blind samples. Therefore, the ability to mask the samples in LIMS is another critical part in creating realistic casework.

### Future Directions

Blind QC programs provide a consistent, objective method by which to continually monitor and improve performance as well as identify errors in the laboratory. This approach also evaluates the entire process from receipt to reporting. Currently, the HFSC blind QC program tracks whether blind cases are detected, and expected results are achieved. As HFSC blind QC sample size increases, the goal is to incorporate error rate determinations. Satisfactory completion is one measure of error, but inter‐analyst differences also provide insight into error rates. Future research involves developing methods to submit the same item to multiple analysts within a section. These error measurements will provide a more accurate approximation of the error rate of evidentiary samples handled by HFSC.

Estimating error rates also requires well‐designed statistical models. Working with statisticians and academic researchers can facilitate these efforts. HFSC is currently working with a group of statisticians to strengthen the blind testing program and more accurately test the system with challenge samples, like those on the brink of sufficiency in latent print analysis, along analytical thresholds in toxicology, or DNA mixtures in forensic biology. Forming these types of collaborations will not only benefit the local criminal justice system, but also provide increased confidence in forensic testing. As more laboratories adopt blind QC programs, an exchange program could be devised to share knowledge, data, and resources. This program could help to reduce the cost and time associated with blind testing and provide more data for error rate determinations.
